# Exploring the experiences of having a child who regularly does not attend school on parental mental health and wellbeing in the United Kingdom

**DOI:** 10.1371/journal.pone.0333501

**Published:** 2025-10-08

**Authors:** Jasmine Chavda, Lauren Denyer, Daksha Trivedi, Amanda K. Ludlow

**Affiliations:** 1 Centre for Research in Public Health and Community Care (CRIPACC), School of Health, Medicine and Life Sciences, University of Hertfordshire, United Kingdom; 2 Department of Psychology, Sport and Geography, School of Health, Medicine and Life Sciences, University of Hertfordshire, United Kingdom; Father Muller Charitable Institutions, INDIA

## Abstract

A significant number of children in the United Kingdom (UK) are counted as regular school non-attendees, with those who are neurodiverse and/or have underlying mental health conditions considered most at risk. Relatively little attention has explored the experiences of having a child who regularly does not attend school on the parents’ own mental health and wellbeing. Thematic analysis was conducted using semi-structured interviews with 11 parents with children who do not regularly attend school in the UK. Two superordinate themes were generated: (1) Pivotal changes to the family dynamic, and (2) Paying a high price. Overall, parents revealed how having a child who did not regularly attend school had negatively impacted their health, wellbeing and their daily life. Many parents acknowledged these experiences had left them needing to access support for their own mental health. These results hold real-world implications for schools, services and health professionals who could provide more personalised support to reduce the negative consequences of school non-attendance. This should be prioritised for children’s overall development and parents’ wellbeing.

## Introduction

In the UK, school non-attendance, also referred to as ‘e*motionally based school avoidance,*’‘*school refusal*’ and ‘*school absenteeism,*’ [1] can be defined by a student being: (a) persistently absent, missing 10% of school sessions or (b) severely absent, missing 50% of school sessions [2]. Since the Covid-19 pandemic, school absences have been one of the most difficult challenges that parents and schools have faced. Statistics from the Department of Education show that 17.79% of students persistently missed school in the 2024−25 autumn term, meaning they missed 10% or more of school [3]. Although this has decreased from 19.44% in the 2023−24 autumn term [3], the Children’s Commissioner, Rachel de Souza, still notes her concerns in the absence enquiry that one fifth of children are still persistently absent from school [4]. School attendance is important for children and young people. Beyond academic achievement, attending school is key to help facilitate social and economic outcomes, peer relationships, support mental health and wellbeing and preparation for a smooth transition into adulthood [5,6]. However, many children and young people struggle to attend school, with data showing that only 5.3% of students have an Education, Health and Care Plan (EHCP) and 14.2% require Special Educational Needs and Disabilities (SEND) support [7]. Failure to provide children and young people (particularly with a neurodiverse condition such as autism) with appropriate support can lead to an increased vulnerability to mental health issues and a risk of school non-attendance [8].

Many children and young people experience difficulties with everyday school life due to the emotional stress it can cause, which may stem from mental health concerns such as anxiety and depression or from a neurodiverse condition [9]. Having a diagnosis of autism and/or attention deficit hyperactivity disorder (ADHD) may increase the risk of school related absence [10,11]. Children with probable ADHD are more likely than neurotypical peers to have poorer attendance levels [12] and school refusal is the most reported reason for school non-attendance in children with autism [13]. Thus, children and young people with SEND may be more vulnerable to school non-attendance [10]. It is important to have effective partnerships between school staff and families and have reasonable adjustments in place for neurodivergent students who experience school non-attendance [14]. However, when this is not in place, it often leads to children leaving the education system altogether, receiving home education and/or alternative provision depending on their circumstances and needs [15].

Schools and local councils have a range of legal powers that can be used if a child or young person is missing from school without a legitimate reason. For example, parents may receive a fine for their child’s persistent absence or if they are considered not to be proactively taking steps to help their child back into school [16]. However, this harsh consequence does not consider the barriers, lack of support and resources for parents to help their children get back into the classroom before enforcing such policy. One example of this is the waiting times for mental health support. Each year more than a ¼ of a million children and young people are waiting for mental health support after being referred to Children and Adolescent Mental Health Services (CAMHS) [17], with large variations in wait times which is often related to the severity of the mental health condition [18]. In addition, the waiting times for support for neurodiverse conditions such as autism and ADHD are rapidly growing due to the large increase in the number of referrals. However, reliable statistical data is unavailable, making it difficult to understand the scale of the challenge at hand [19].

Another example is that the issuing of EHCP’s, which is not only a complex process but also comes with lengthy wait times. Some children and young people have been found to wait for more than a year for an EHCP, meaning they go a whole academic year without the vital support they need [20]. Importantly, delays in accessing an EHCP have been shown to have significant repercussions for children and young people’s mental health and wellbeing. For example, children who successfully obtain an EHCP compared to those still waiting for one, have been shown to experience less deterioration in their mental health [21]. However, the impact on parental health and wellbeing cannot be ignored. A qualitative study exploring mother’s experiences of accessing an EHCP for their autistic child, found that mothers reported the process overwhelming and difficult, and revealed a sense that the system was setting them up to fail, resulting in high levels of anxiety and hopelessness in how to help their child [22].

The current study was rooted in the Stress Process Model, developed by Pearlin [23], which provides a theoretical framework for understanding the care stressors experienced by individuals providing care for other family members. With reference to parenting, it is the extent to which the stressors of the parenting role itself (e.g., time, effort and energy) affect other aspects of their life (e.g., work, relationships, family and leisure) [24], where increased stressors can lead to poor mental health outcomes [25]. Previous research has highlighted that parents of children and young people with mental health and/or neurodiverse conditions generally have more parenting difficulties [26,27], which is associated with higher levels of caregiver burden and poor mental and physical health [28,29]. Many parents of these children are also often unable to work but may have multiple children to provide for and meet the needs of. For example, even sending one child to state school has shown to cost a minimum of £864.87 a year for a primary aged child, and £1,755.97 a year for a secondary aged child [30]. Therefore, making it increasingly difficult for parents to financially support their children in school. This is just one example where the burden of care may overspill into other aspects of parents’ lives, which in turn may have an impact on their own mental health and wellbeing. Understanding parental wellbeing is vital, not only for the parent themselves, but their children, family and wider social and societal networks [31].

Some research has explored school non-attendance, including insights into child, parental and school perspectives, plus how school non-attendance is associated with poor mental health and neurodiversity of children and young people themselves. However, there is currently no research to date that explores the experiences beyond this. Therefore, this qualitative study aims to interview parents of children who do not regularly attend school, to gain new knowledge and understanding of their experiences and their perceptions of how this has affected parental mental health, wellbeing and their lives. The research questions aim to address the following: (a) What are the experiences of parents whose children do not regularly attend school? (b) How have these experiences affected parental mental health, wellbeing and quality of life?

## Materials and methods

A qualitative exploratory design was chosen to capture the voices and lived experiences of the parents in this study. Using thematic analysis enabled the researchers to explore, in depth, parents’ experiences of having children who do not regularly attend school and the impact on their own mental health and wellbeing. And, to use a reflexive approach for a detailed understanding of the codes and themes within the data [32].

The interview schedule for the qualitative interview was developed using open questions to encourage discussions around their child’s school non-attendance, the support they received and the impact this had on the wellbeing of themselves and their family to date. Parents were asked at the beginning of the interview which terminology they preferred with regards to their child’s school non-attendance, as the different terms (e.g., school refusal, school anxiety, school avoidance) tend to be used interchangeably. ‘School-absenteeism’ was generally preferred to remove the blame and choice of not attending school from the child. Therefore, this was used as per the interview schedule below but was adapted during the interviews where necessary. The questions included:

1. Can you give me a brief overview of your experiences of your child’s school absenteeism?
2. Can you tell me about the support you as a parent/carer received regarding their school absenteeism?
3. Can you describe the impact the level of support you were given as a parent/carer had on your relationship with your child and your relationship with your child’s school?
4. How did your child’s absenteeism affect life at home?
5. How did your child’s absenteeism and the process of seeking support affect you and your own mental health, and did you seek any support for yourself?
6. Are there any self-help strategies you have used to help to support your own mental wellbeing?
7. Can you describe the impact the process has had on you and your family? Any positives?
8. How do you think the process could be improved to better support parent/carer wellbeing?
9. Do you have any advice for parents/carers going through this process on how they could support their own mental health and wellbeing?

### Participants

A purposive sampling method was used to recruit parents/carers of children aged 7−16 years who regularly do not attend school or had regularly not attended school in the past and were recruited using a snowball effect. Advertisements containing information about the study were shared on X, Facebook groups and with local charities and organisations (recruitment period: 22/02/2024–30/09/2024). Parents were asked to contact the lead researcher via email if they were interested in taking part.

Fourteen parents were originally contacted on a first come first served basis in response to them signing up to take part in the study. From these, 11 parents gave consent and were interviewed. Interviews lasted from 24.02–77.31 minutes (*M* = 47.69, *SD* = 15.88). The 11 parents (9 mothers and 2 fathers) were aged 33–56 years (*M* = 45.45, *SD* = 7.19) and most considered their ethnicity to be white British (*n* = 8) with the remaining individuals identifying as white European, Asian and Black respectively (*n* = 3). They were from a range of different geographical locations but all falling within the UK. Participants’ children were school non-attenders and were aged 8–15 years (*M* = 12.18, *SD* = 2.86) and their school non-attendance varied in time and frequency. Of the 11 children, 8 had an identified neurodiversity and/or mental health condition and 1 had a physical health condition. The demographics for parents and their children can be seen in Table 1.

**Table 1:** Characteristics of parents and their children.

PPT ID	Parent age	Relation-ship to child	Marital status	Geographical location	Ethnicity	No. of children and ages	Age of absent child	Gender of absent child	Length of time child missing from school	Identified neurodiverse or mental health conditions	EHCP?	Primary or secondary school	Type of school
**1**	33	Mother	Cohabiting	Mansfield	White	Two: 8, 9	8	Male	Varied on and off	Autism and undiagnosed anxiety	No (collecting evidence)	Primary	State
**2**	42	Mother	Married	Lincolnshire	White British	Three: 9, 13, 23	13	Male	18 months	None	No	Secondary	State
**3**	47	Mother	Separated	Isle of White	White British	Two: 12, 14	12	Male	3 years	No	Yes	NA (Deregistered)	NA (Deregistered)
**4**	53	Father	Cohabiting	Manchester	White European	One: 15	15	Female	2 years	Autism	Yes	Secondary	Independent
**5**	36	Mother	Married	Bedfordshire	White British	Two: 1, 7	7	Female	2 months	Speech and language disorder	No	Primary	State
**6**	46	Mother	Married	Reading	White British	One: 11	11	Female	4 years	No	No	Primary	State
**7**	41	Father	Married	Bedfordshire	Black	Two: 8, 15	15	Female	Frequent	Autism	Yes	Secondary	State
**8**	47	Mother	Cohabiting	Milton Keynes	White, Caucasian	Two: 13, 15	15	Male	2.5 years	General anxiety disorder	Applying	Secondary	State
**9**	53	Mother	Married	Leicestershire	Asian	Two: 10, 11	10	Male	70% attendance	ADHD, Autism, anxiety	Applying	Primary	State
**10**	56	Mother	Single	Surrey	White British	Two: 14 (twins)	14	Male	4 years	ADHD, PDA and dyslexia	Yes	Secondary	State
**11**	46	Mother	Single	Sheffield	White British	Three: 14, 10 (twins)	14	Non-binary	Over 1 year	Autism (detained and sectioned)	Applying	Secondary	State

### Procedure

After signing up and being invited to take part, 11 parents were emailed a copy of the participant information sheet, the informed consent form, and a copy of the interview schedule before consenting to take part. Once written consent had been obtained, parents arranged a time for the interview to take place. Since the interview was taking place online via Zoom the parent was able to choose a location and time convenient to them as long as it was a quiet and private place. The lead researcher conducted all the interviews, and a £25 e-voucher was emailed to each parent for their participation.

### Ethical considerations

Approval was awarded by the University of Hertfordshire Ethics Committee (original protocol number: LMS/SF/UH/05569) and this study was conducted in accordance with the Declaration of Helsinki. Parents were provided with sufficient information about the study to facilitate providing their informed consent. Parents were made aware that they would be recorded and how the recordings would be saved, stored and deleted upon transcription. Confidentiality was adhered to throughout the study. During transcription, identifiable information was removed. Each parent was given an ID number which is used throughout this report. Parents were provided with a debrief sheet including supportive websites following the interview.

### Data analysis

A reflexive thematic analysis was performed enabling both recurring ideas as well as diverse impact of school non-attendance on the families to be identified and explored [32,33]. Once transcription was complete, the lead researcher (JC) conducted the thematic analysis, whereby transcripts were read and re-read, and initial themes were identified from the codes. This process was then conducted by AL. Reflexive conversations between these two authors were undertaken to refine the themes identified and to create the theme table with supporting quotes [32], with support from the research team so that the transcripts and codes could be discussed (LD and DT). 

Direct quotes from participants are presented below in italics, with the interviewee’s referred to as parents. Square brackets indicate substituted words needed to maintain anonymity to the quotation. Where there is a break in the text presented from the direct quotation, this is denoted by ‘…’.

## Results

**Fig 1:**
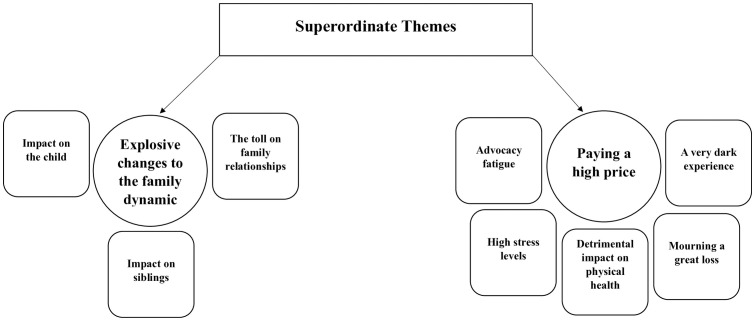
Superordinate themes and their sub-themes.

Following the thematic analysis**,** the interviews resulted in the following superordinate and sub-themes as seen in Fig 1 below.

### Superordinate theme 1: Pivotal changes to the family dynamic

This theme explores how the experiences of having a child who does not regularly attend school affects the wider family unit, which consequently has a negative impact on parents’ lives.

#### Sub-theme 1: Impact on the child.

There was a strong consensus among parents that the distress their child experienced due to their struggles with the school environment had serious physical and psychological consequences. For example, Parent 1 and 4 reflected on how the experiences had changed their children.

*“He was having panic attacks and then, physically unable to move. He was feeling sick. He was having diarrhoea every night” … “he’s gone from being this happy boy to this shell of a child that I don’t even recognize.*” (Parent 1)

*“She went into the 1st 3 days of school, and then she stopped, and then she just said, ‘I can’t. I can’t do this anymore. I can’t. I can’t do it.’ So, she effectively just went into sort of collapse and burnout at that point in time.”* (Parent 4)

Similarly, Parent 2 revealed the following about their child*, “Couldn’t eat. He couldn’t sleep. I was having to sleep in his bed with him. Just so and hold him just so that he’d relax because he was so distressed.”* They then went on to disclose the extent of its impact, *“he wanted to die. And that school was like a prison, and he’d rather be dead.”*

For one child, their parent revealed that they really did want to be in school but could not cope with the school environment anymore.

*“He wasn’t enjoying it. It wasn’t fun to not be happening, you know. He was distraught by the fact that he couldn’t cope, and that he couldn’t be in school because he wanted to be there.” “… he just couldn’t cope. It was like he was traumatized he would just shut down completely. You know, either stop talking or just freeze on the spot, basically.”* (Parent 3)

Another parent revealed other consequences of their experiences on their child, *“was self-harming quite significantly and had stopped eating,”* (Parent 11).

These sentiments show the extent to which accessing the school environment is impacting these children both psychologically and physically. It also shows the distressing behaviour and situations parents are having to deal and cope with. This in turn had an impact on other members of the family.

#### Sub-theme 2: Impact on siblings.

Parents who also had other children revealed that these children generally found the school environment accessible and attended regularly. However, they made it clear that the experiences and consequences of their child who experienced school distress and did not regularly attend, still had a negative impact on their other children. For example, many siblings found it difficult to understand why they still must go to school, *“she’s only 9, almost 10. She doesn’t quite understand why [insert child name] gets days off and she doesn’t,”* (Parent 1). Particularly when the child who does not regularly attend school seemingly has the freedom of choice to stay at home and do as they please.

“*And it was very hard for him to manage at school for a while and go in when his brother was at home gaming and, eating whatever he wanted in the middle of the night,”* (Parent 10).

*“But I think there are definitely days where she doesn’t really feel like school and knowing her brother’s just sitting at home on the computer all day. That’s gotta be hard for her.”* (Parent 3)

This appears to cause a lot of frustration for the sibling, “*And for my son he’s had, he was very angry and very, but not aggressive,”* (Parent 10), and arguments, *“it’s a learning curve for everybody every single day about it erm, so it’s really hard, and it does cause quite a lot of arguments, I won’t lie.”* (Parent 1). Furthermore, due to the additional caring responsibilities, parents have less time for their other children (and often less able to work) and recognised how this affected their lives.

*“so like the normal things I do around my job like be going to little assemblies. I’d be going in and doing reading like all those things stopped. I stopped being able to take him to school erm, so that was a big hit for him as well.”* (Parent 1)

*“There’s also the fact that because I can’t work financially, things are more difficult.”* (Parent 3)

Parents also revealed a real sense of worry for the unknown impact this would have on their other children in the future including their relationship dynamic.

“*She’s missed out on stuff already, and she’s 1 years of age because of meeting her sister’s needs. So, we are having to deeply look at how we do things. So that we make sure that her needs are being met, and she doesn’t turn into the glass child,”* (Parent 5).

*“I suppose I won’t really find out how the twins experienced this until probably quite a lot later. Guess at the moment they’re just rolling with it because it’s their version of normal to have a sibling with significant mental health difficulties*…*that will probably come back to bite me when they hit their teens and realize how compromised a lot of things were.”* (Parent 11)

These sentiments highlight the burden of having a child who does not regularly attend school, and the difficultly and the sacrifices being made, as they *struggle* to meet *all* their children’s needs.

#### Sub-theme 3: The toll on family relationships.

There was a consensus among parents that having a child who does not regularly attend school is challenging, with the impact extending throughout the whole family, *“it has really been a stressful journey for everyone member of the family,”* (Parent 7). One parent gave their insight as to why:

“*Don’t underestimate the impacts that it has on the whole family …. to think that it’s just Monday to Friday 9, till 3, it’s not. It affects every aspect of your life of the children’s lives of your life. I think when you’re really vulnerable, children are really vulnerable, and you, as parents, are really vulnerable.”* (Parent 2)

Parents went on discuss the impact on their own romantic relationships, where one parent disclosed that they were being spread so thinly catering to the differing needs of their children. This meant they were unable to meet the needs for fulfilling romantic relationship, which contributed to its breakdown. “*I’m now divorced or getting divorced, and I’m sure that the stress of the situation contributed significantly to that…Because I was so focused on trying to help the kids that I had nothing left to give my husband. He was so intolerant of what was going on, that it created an adversarial situation between me and him.”* (Parent 3)

Similarly, Parent 2 revealed *“the emotional toll that it’s taken on me and my husband has been, it really could have gone one of 2 ways you know.”* They expressed it was down to the strength of their relationship that carried them through their experiences but understand how damaging it could be for others. *“I think if we hadn’t been as strong as we were before it could have easily affected our marriage … But fortunately, we pulled together rather than apart. But I can absolutely see how it just rips family’s apart.”*

In addition, the impact felt from both sides of the wider family cannot be ignored, with the grandparents from either side being mentioned; *“it’s really caused the ridge between myself and my parents,”* (Parent 8), and *“But I mean my husband’s sides of the family … we’re barely invited to anything other than the odd birthday meal here and there.”* (Parent 5)

These sentiments demonstrate the high price parents are paying, and although they are trying to manage, they are struggling with the demands of their children and meeting their needs while still maintaining strong relationships with their romantic partners and the wider family. They also highlight their worries surrounding the new family dynamic.

### Superordinate theme 2: Paying a high price

This theme explores how parents appear to be paying a high price because of the complexities surrounding their child being unable to regularly attend school. Their experiences reveal the negative impact this has on their stress levels, their physical and mental health, their identity, and their everyday life.

#### Sub-theme 1: Advocacy fatigue.

Parents reported a need to remain strong for their child who is unable to attend school, their other children and their families, and there was a sense for the need to fight and advocate for their child, *“I have to be the voice that gets heard because his voice won’t get heard,”* (Parent 1). Which was expressed as being part of the parental role*, “it’s hard but you’re a mom, that’s what you do”* (Parent 1), and *“I think I just kind of get on with. You kind of have to get on with it when it’s your kids. You can’t really have a choice”* (Parent 3). However, the consequences of this revealed by parents, show the toll it has on other aspects of their lives.

#### Sub-theme 2: High stress levels.

There was an overwhelming sense that the experiences and complexities surrounding of having a child who does not regularly attend school has left many parents feeling overwhelmed, *“I definitely was traumatized by all of this,”* (Parent 10), and physically upset, *“I have a little cry every now and again,”* (Parent 1). Which has led to higher stress levels and therefore had a detrimental impact on their mental health and wellbeing. *“It’s stress, isn’t it?... You do end up going to dark places. But then, I don’t know, you just come out of it … I would say, stress levels are on about 98-99% all the time … I’ve started having all these serious medical conditions in this last month. They’ve all appeared because of the stress.”* (Parent 9)

Parents went on to reveal feelings and symptoms that more closely resembled depression and anxiety. These were discussed in relation to themselves, how their partners were affected, and the services they have been seeking in a bid to cope.

*“So, both myself and my husband definitely spiralled into anxiety and depression. Last year, before I got pregnant with the baby, I got actually got signed off from work for anxiety … my husband spiralled into deep depression, and he’s still clawing out of it now shall we say. I think the only reason why I’m not literally on the floor kicking and crying myself is because I go to work. I have other things, and I make sure we go out and we try and go on the positives. I’ve gone through counselling personally myself... it’s been a struggle,”* (Parent 5).

These sentiments reveal how increased levels of stress are affecting parents’ mental health and wellbeing, leading to the potential development of mental health conditions.

#### Sub-theme 3: Detrimental impact on physical health.

There was also an overwhelming sense that parents’ physical health had been impacted as a result of a decline in their mental health, *“I’ve been feeling the physical effects of stress definitely,”* (Parent 6).

*“We’re all absolutely exhausted* … *you know but like the physical energy it’s just gone like everything was just taken up with us giving everything we can to [insert child name],”* (Parent 2).

One parent even revealed how their poor mental health was having a direct detrimental impact on their physical health, *“but I feel depressive symptoms, and most times it comes with migraines and headaches,”* (Parent 7).

Others revealed how the overall stressful experiences had negatively affected their own physical health, *“I am a very aware that there’s you know, the some of the aches and pains and things I’m feeling is probably my body just dealing with the stress and the frustration,”* (Participant 6), and that of their partner, *“as I say, my husband again, naturally, from being more sedentary because you know, even with the best will in the world, and going out for walks and stuff. We are exercising less. And he’s of that age that yeah, he’s his cholesterol has gone up so. But he’s got that under control. But yeah, but I said, that’s a direct impactive,”* (Parent 6).

These sentiments highlight that the fatigue involved in advocating for, and the burden of caring for a child who does not regularly attend school can increase parents’ stress levels, which has a direct impact on their psychological and physical wellbeing. And while they may have been revealed as individual concerns, overall, the impact creates a complex interaction between mental health concerns and physical health concerns.

#### Sub-theme 4: Mourning a great loss.

During the interviews there was an overwhelming sense that parents were suffering a great loss in many aspects of their lives. This included a sacrifice of their lives, their careers, their relationships and identity; there was a consensus among many of the accounts.

*“so, you could kind of argue I’ve lost my* career*, my husband, you know my marriage. As a result of that, we now have to sell the family home. So, we are going to lose our house, you know. So, it has had a huge impact on the life we were living.”* (Parent 3)

Similarly, another parent revealed the impact on their financial situation, *“we’ve been skint because we’ve been missing out on my wage, so we’ve missed out on being able to do stuff,”* (Parent 2)

Another parent also lost their career, which had financial implications for their family and their own mental health.

*“There have been big negatives … I had to give up my* career *… and now I live on benefits because it was just impossible to work and juggle all of that stress. So, that’s been massive for me personally, for my mental health as well as you know, the financial impacts on the family, because I liked my job, I liked my* career*. And now I feel very much like, I’ve just might become a carer and that is hard, and it’s hard because you can never let them know that that’s an impact if that makes sense. So, you have to kind of act like it’s not a big deal when it actually is!”* (Parent 3)

*“To go from earning 3,000 pounds a month to 300 pounds a month, you know, for like for 3 years, see what impact has on the mental health.”* (Parent 4)

Parents went on to describe how their life had not panned out as they had hoped it would as they aged as a parent with a family.

*“But you know that’s it’s it has been very, very, you know, very difficult, because it’s, you know, the life that I envisage that would have, you know, early fifties, you know sort of hopefully winding your career down, building your pension up, you know all of all of that has gone now for me, so that you know that has that has been very challenging.”* (Parent 4).

These sentiments reveal a sense of a great loss that the parents are experiencing in their lives, negatively impacting their financial stability and consequently their own mental health.

#### Sub-theme 5: A very dark experience.

Parents also expressed a sense of darkness surrounding the demands placed on them as parents of children who do not regularly attend school. These were revealed as a result of discussing the high price they are paying and the impact on their own health and own life. *“I’m signed off work erm, because I can’t! I just can’t! It’s been really, really the most challenging year of my life, and it’s taken a massive, massive toll on me. The doctor’s aware. I’ve felt suicidal at times. It’s everything just has gone crashing down,”* (Parent 11).

Parents went on to describe how scary and unstable their lives felt*. “It’s horrible. It’s just terrifying,”* (Parent 11).

*“For the 2 years before he was off permanently it was absolute hell. My life was 24 hours a day, hell. You know, I’d go to sleep not knowing how it was going to be in the morning. I’d wake up, not knowing if I was going to get him into school and in between it was horrendous. If he was at school, it was horrendous, and if he wasn’t at school, it was horrendous,”* (Parent 10). One parent even referred to their lives as being a complete nightmare, showing the dark feelings they have towards the impact their experiences have on their lives, *“Like I say it’s a nightmare, complete nightmare. It just takes over your whole life.”* (Parent 9). Similarly, one parent revealed, *“It’s been a horrific experience. I think it’s probably the most difficult thing that me and my partner have been through in our whole time on this earth I think,”* (Parent 8).

These sentiments highlight the extent to the challenges surrounding having a child who is unable to attend school has impacted their entire lives, and the experiences of dark thoughts and feelings for so many of these parents.

## Discussion

This study aimed to explore the experiences of parents whose children do not regularly attend school and identify how these experiences affected their mental health, wellbeing, and quality of life. Their experiences highlight how the burden of having a child who does not regularly attend school not only affects the child in question, but the stress and demands on parents negatively affect siblings and the wider family circle. This is particularly apparent if the child has additional needs. The results from this study found there were negative consequences for marital relationships, where a breakdown in a marriage could have negative consequences for parental mental health, contributing further to the stress they have been experiencing [34]. Being happily married is associated with better physical and mental health and promotes better wellbeing due to access to resources including both social and economic [35]. However, parents revealed that they were being spread so thin to meet the needs of all their children, let alone meet the needs of their spouses regardless of martial support. Thus, this shows support for the Process Stress Model in relation to parenting [23] where the added stress of their parenting role affects other aspects of their life [24], leading to poor mental health [25].

Parents in the current study also felt they had less time, money and energy to fulfil the needs of their other children, causing anger and frustration. Children who have siblings that have additional needs, for example, a neurodiverse condition, are often overlooked and their feelings are minimised [36]. This may have negative consequences for their self-esteem and future relationships if their parents are not able to express the same level of love and support [37]. Parents revealed a sense of worry about the longer-term impact on siblings’ health and wellbeing, adding to their already high stress levels. This demonstrates that the burden of care can have a detrimental effect on parental health and wellbeing.

Furthermore, parents of children and young people with mental health and or neurodiverse conditions generally experience more parenting difficulties [26,27], which is associated with higher levels of caregiver burden and poor mental and physical health [28,29]. Since many of the parent’s children in this study have neurodiverse and/or mental health conditions, this reinforces the negative impact beyond the child in question and shows the impact on the wider family’s health and wellbeing, when appropriate support is missing. Parent’s wellbeing and mental health play a vital role in providing the necessary care of their children [38] and also for their own life satisfaction [39], showing that these parents require better support for their own health and wellbeing, to better meet the needs of their children, and better meet their own needs.

Parents reported that the high levels of stress in their lives had a direct impact on their physical health, such as headaches, migraines, aches, pains and exacerbated current health conditions. Moreover, the lack of time for themselves meant there were less opportunities to exercise or look after themselves thus increasing risk of weight gain and related concerns. There is an association between physical health and mental health [40], whereby physical health problems can increase the likelihood of developing mental health problems and vice versa [41]. Again, these results show how the greater demands on these parents increase stress, affecting other aspects of their lives and negatively affecting their mental health and wellbeing, providing further support for the Stress Process Model [23].

Parents also highlighted the sacrifices they experienced because of their child being unable to regularly attend school. For example, many parents reported having no choice but to give up their work to care for their children. This firstly meant giving up their careers that they enjoyed and gave them purpose without knowing when they would be able to return to work. Having a career break to care for children has negative consequences for future career prospects particularly for mothers [42], which may cause future burden on parental mental health and wellbeing. Secondly, giving up work meant that parents were also dealing with the financial implications of a loss of income, which may impact their ability to be able support their child back into school. It is costly to send your child to school, and parents with a significantly reduced income and multiple children to provide for may not financially be able to support their child back into school [30]. In addition, lifestyle adjustments such as moving house and sacrificing their basic needs increases levels of stress and has negative consequences for parental health, wellbeing and quality of life for themselves and the whole family. This added pressure for parents and families in already stressful circumstances, shows that parents need additional support to be able to get their child back into school, and to increase their wellbeing.

The findings from this study are an important step into exploring the experiences of having a child who does not regularly attend school on parental mental health and wellbeing. Since the Covid-19 pandemic, school absences have been one of the most difficult challenges that parents and schools have faced. While recent figures from the Department of Education have shown a slight decrease in persistent and overall absence rates, severe absence rates have increased in primary, secondary and special schools [3]. This reveals that appropriate training and timely support is needed. The government need to prioritise mandatory teacher training for schools to: (a) provide better support for parents with a child who is unable to regularly to attend school, and (b) provide the necessary resources and support to encourage children back into the classroom. More rigorous SEND training for schools would also be beneficial to: (a) enable teachers to identify different types of SEND in the classroom, and (b) have access to early intervention to support younger children’s readiness, inclusive of sensory processing, motor and visual perceptual skills [43]. As this early support and intervention may be beneficial for keeping children in schools, reducing the care burden on parents, thus less likely to negatively affect their mental health and wellbeing. However, since these implications are real for the parents currently living through the experiences discussed, mental health support for them would be of great benefit; and to prevent a decline in their mental health and wellbeing. Government or council initiatives should be developed to support parents with their children who are unable to attend school, rather than penalise them without understanding the challenges at hand.

It is important to note some of the strengths and limitations of this study. The fact that a wide geographical location in the UK is covered by the participants is a strength, despite there being a small sample. In addition, the findings capture the voices of parents with lived experiences, and how their lives and family dynamic are affected by their child being unable to regularly attend school, who may also have additional needs such as a neurodiverse condition. Although it is recognised that a limitation of qualitative studies is their generalisability, this research still contributes to the landscape of school non-attendance. However, some factors may have influenced the generalisability of the results. The use of snowball sampling and the choice of recruitment through parent support groups, may have led to an over-representation of mothers from White British backgrounds in the sample. Future research should include wider samples of the population to understand more about the experiences of different groups (e.g., mothers vs fathers) in addition to the differences between cultures, and type of school (e.g., primary vs secondary and state vs private). Furthermore, many of the parents in the current study still had a child who was regularly missing school, and it would be important for future studies to also capture the voices of those who were now accessing some form of education, whether this be through alternate provision, home schooling or returning to school.

The findings also reveal the need to explore the longer-term impact of school non-attendance on the health and wellbeing of families affected. Research could also explore the development of the child unable to attend school and their siblings, and the role of neurodevelopmental disorders. It would also be useful to understand the economic implications with additional services being required to support the health and wellbeing needs of parents and their families.

Overall, being a parent of a child who does not regularly attend school is a challenge, both physically and mentally. The parents in this study highlight that the increased stress from parenting demands, in addition to the lack of support from schools, services and family members does negatively affect their mental health and wellbeing. Although parents have expressed that it was their job to advocate for their child and support them as best they could, the advocacy fatigue shows that parents need support themselves to combat the negative consequences it is having on them and their family. The results of this study hold real-world implications for schools, services and health professionals who could provide more personalised support for children and families to reduce the negative impact of school non-attendance. This should be prioritised for children’s overall development and parental and family health, wellbeing and quality of life.
